# Radiofrequency Schottky Diodes Based on p-Doped Copper(I) Thiocyanate (CuSCN)

**DOI:** 10.1021/acsami.1c22856

**Published:** 2022-06-01

**Authors:** Dimitra G. Georgiadou, Nilushi Wijeyasinghe, Olga Solomeshch, Nir Tessler, Thomas D. Anthopoulos

**Affiliations:** † Electronics and Computer Science, University of Southampton, Highfield Campus, Southampton SO17 1BJ, United Kingdom; ‡ Department of Physics, Imperial College London, Prince Consort Road, South Kensington, London SW7 2AZ, United Kingdom; § The Sarah and Moshe Zisapel Nano-Electronic Center, Department of Electrical Engineering, Technion-Israel Institute of Technology, Haifa 3200, Israel; ∥ King Abdullah University of Science and Technology (KAUST), Division of Physical Sciences and Engineering, Thuwal 23955-6900, Saudi Arabia

**Keywords:** p-type diodes, high-frequency rectifiers, RFID, NFC, molecular doping, nanogap electrodes

## Abstract

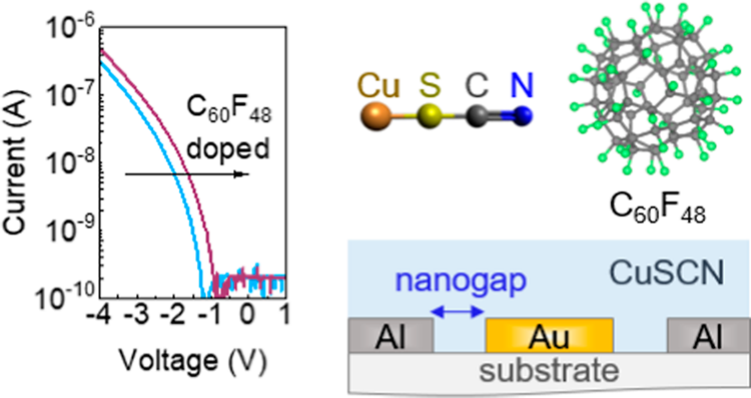

Schottky diodes based on inexpensive materials that can be processed using simple manufacturing methods are of particular importance for the next generation of flexible electronics. Although a number of high-frequency n-type diodes and rectifiers have been demonstrated, the progress with p-type diodes is lagging behind, mainly due to the intrinsically low conductivities of existing p-type semiconducting materials that are compatible with low-temperature, flexible, substrate-friendly processes. Herein, we report on CuSCN Schottky diodes, where the semiconductor is processed from solution, featuring coplanar Al–Au nanogap electrodes (<15 nm), patterned via adhesion lithography. The abundant CuSCN material is doped with the molecular p-type dopant fluorofullerene C_60_F_48_ to improve the diode’s operating characteristics. Rectifier circuits fabricated with the doped CuSCN/C_60_F_48_ diodes exhibit a 30-fold increase in the cutoff frequency as compared to pristine CuSCN diodes (from 140 kHz to 4 MHz), while they are able to deliver output voltages of >100 mV for a *V*_IN_ = ±5 V at the commercially relevant frequency of 13.56 MHz. The enhanced diode and circuit performance is attributed to the improved charge transport across CuSCN induced by C_60_F_48_. The ensuing diode technology can be used in flexible complementary circuits targeting low-energy-budget applications for the emerging internet of things device ecosystem.

## Introduction

Over the last decade, there has been a growing interest in delivering electronics that are flexible, lightweight, disposable, low-power, and inexpensive to be used in wearable devices and as integral part of the internet of things ecosystem.^1^ A number of electronic devices, such as light-emitting diodes, solar cells,^2^ sensors, microprocessors,^3^ and supercapacitors, have been demonstrated on flexible substrates.^4^ All these components need to be integrated with hardware that provides wireless connectivity and/or radiofrequency (RF) energy harvesting capabilities to render them truly energy-autonomous and connected. To this end, significant progress has been made toward scalable manufacturing of flexible diodes and rectifiers that can harvest RF signals from ambient sources or from a reader and convert them to usable direct current (DC) signals, which can then be used to power the microelectronic components.^5^ Of particular relevance to commercial applications are RF identification (RFID) and near-field communication (NFC) technologies that operate at low (125 and 135 kHz) and high (13.56 MHz) frequencies to enable object identification and peer-to-peer data transfer usually via mobile phones.^6^

The operational frequency of the rectifier is dictated by the diode performance, which depends both on the semiconductor material’s electronic (transport) properties as well as on the device geometry and architecture. In general, large-area processable n-type diodes outperform their p-type counterparts as the electrical performance of the latter is limited by the intrinsically lower (hole) mobility, which is in turn set out by the scarcity of suitable materials. Furthermore, the materials used must comply with the low temperature processing (<200 °C) constraints posed by the inexpensive flexible substrate. This further narrows the gamut of materials that can be employed.^5^ Organic materials, such as small molecules and polymers, have been the materials of choice for RF diodes due to their ease of processing using solution-based and printing methods,^7^ although higher (>GHz) cutoff frequencies have been reported with vacuum-deposited small organic molecules,^8^ metal oxides,^9^ Si microparticles,^10^ and low-dimensional (2D) transition metal dichalcogenide materials.^11^ The main drawback of all these materials, however, is the very high cost, associated with either their synthesis and purification (organics) or their integration with high device yield in large-area manufacturing processes (e.g., 2D materials).

Herein, we propose a route toward improving the performance of Schottky diodes based on the inexpensive inorganic molecular p-type semiconductor copper(I) thiocyanate (CuSCN), which is processed from solution at very low temperatures (80–100 °C), thus compatible with the majority of targeted flexible and biodegradable substrates (e.g., plastic and paper).^12^ We apply a two-pronged optimization approach that includes both material and device engineering. More specifically, we increase the mobility of CuSCN via molecular doping using the molecular p-type dopant C_60_F_48_ and employ a coplanar high-aspect-ratio nanogap device architecture, fabricated using the high-throughput adhesion lithography (a-Lith) technique.^13^ We demonstrate p-doped CuSCN/C_60_F_48_ diodes and rectifiers that operate at frequencies up to 13.56 MHz and deliver hundreds of millivolts of output voltage, capable of powering the internet of tiny things, which includes microelectronic devices with minimal power budget (e.g., an RF-based wakeup receiver).^14^

## Experimental Methods

### Fabrication of Nanogap Electrodes

Coplanar nanogap separated Al–Au electrodes were patterned using a-Lith following previously reported procedures.^15^ In brief, an aluminum (Al) electrode with a thickness of 40 nm was thermally evaporated in high vacuum (10^–6^ mbar) on 2 × 2 cm Borofloat glass substrates and patterned to the desired design using photolithography, followed by wet etching. The patterned substrates were soaked in 1 mg mL^–1^ solution of octadecylphosphonic acid (Sigma Aldrich) in isopropanol to form a self-assembled monolayer (SAM) on the Al surface. Next, a global gold (Au) electrode (35 nm) with a 5 nm Al adhesion layer was sequentially deposited via thermal evaporation. An adhesive film (First Contact, Photonic Cleaning Technologies) was applied on the top of Au and left to dry in air. Then, the adhesive glue was peeled off, removing the Au metal from the top of the SAM-functionalized Al. The SAM and any remaining photoresist were cleaned via 10 min ultraviolet (UV)–ozone treatment to reveal an empty nanogap typically on the order of 10–15 nm.

### CuSCN and C_60_F_48_-Doped CuSCN Formulations and Film Deposition

Cu(I)SCN powder (Merck, 99%) was dissolved in diethyl sulfide (DES) (Merck, 98%) at concentrations of 5 mg mL^–1^ and stirred for 1 h at room temperature. C_60_F_48_ was synthesized, as described in refs (16) and (29) at the Jozef Stefan Institute (Slovenia), and the purity was estimated to be 96%. C_60_F_48_ powder was dissolved in DES at room temperature, and then, calculated volumetric quantities of C_60_F_48_/DES (μL) were added to the CuSCN/DES solutions to create dopant concentrations of 0.05–1 mol %. Next, the C_60_F_48_-doped CuSCN precursor solutions were spin-cast at 800 rpm for 60 s in a N_2_-filled glovebox, and the films were annealed at 100 °C for 15 min.

### Thin Film Surface Characterization

Optical microscopy images were captured using a Nikon Eclipse E600 POL optical microscope. The scanning electron microscopy (SEM) topology images of Al/Au nanogap electrodes were acquired using an ultra-high-resolution field emission Magellan scanning electron microscope equipped with a two-mode final lens (immersion and field-free).

### Electrical Characterization

Current–voltage (*I*–*V*) characterization of the diodes was carried out on an Agilent B2902A parameter analyzer at room temperature inside a N_2_-filled glovebox. The measurement resolution of this source measure unit is 100 fA. High-frequency measurements were performed by applying an alternating current signal to the circuit shown in Figure 5a using an Agilent 33220A function generator. This was achieved by mounting a 1 nF capacitor directly onto the measurement micromanipulator, while the output voltage was measured across a load resistor *R*_L_ = 1 MΩ. A photograph of the experimental setup is shown elsewhere.^17^ The output and input signals were monitored using an Agilent DSO6014A oscilloscope. The setup was controlled using LabView program.

## Results and Discussion

**Figure 1:**
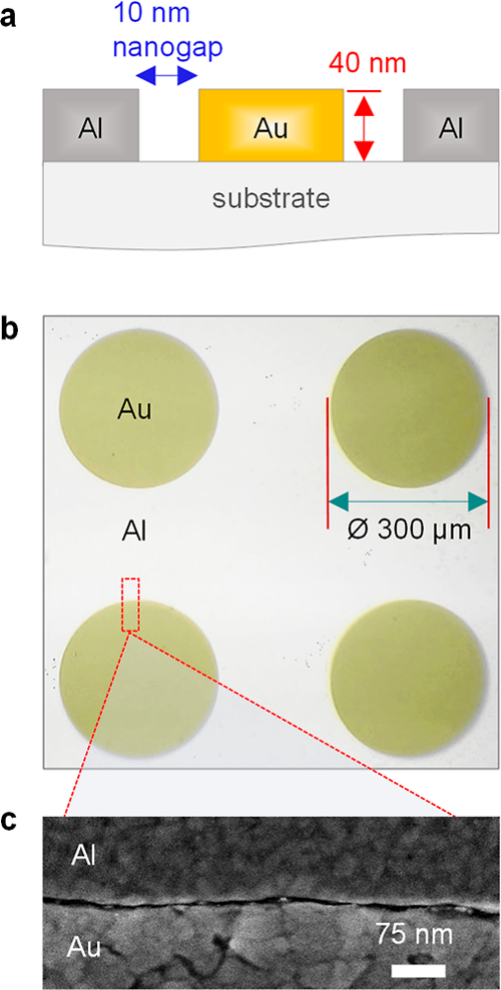
(a) Schematic of the electrode cross-section, depicting the 10 nm nanogap separating the two metals, formed via a-Lith. (b) Optical micrograph of the coplanar Al–Au electrodes with a diameter of 300 μm, where the circular shape was patterned via photolithography. (c) Top view of the nanogap along the metal electrode interface imaged using SEM.

The coplanar Schottky diodes were fabricated with circular patterns of asymmetric Al–Au electrodes (Figure 1a,b). The width of the diodes (*W*) was defined by the circumference of the circles bearing a diameter of 300 μm (diode width: 942 μm), as shown in the schematic in Figure 1b, while the two metals were separated by a 10 nm nanogap channel length (*L*). The high-resolution SEM image in Figure 1c shows the ultrashort and homogeneous gap that is formed along the two metals. a-Lith has been proven to yield consistent nanogaps in the range of 10–15 nm.^15^ Given these geometrical values, the aspect ratio of the diodes (*W*/*L*) is around 10^5^. This geometry is advantageous to commonly employed vertical structures when it comes to high-frequency applications. A high-aspect-ratio coplanar structure allows for considerable resistance and capacitance reduction by confining the active material to the nanogap channel length, while the device width remains on the macroscale, thus overcoming film deposition challenges (e.g., pinholes) often encountered with ultra-thin films. Using such coplanar nanogap device structures, we have demonstrated high-frequency (13.56 MHz) diodes based on n-type semiconductor zinc oxide (ZnO) processed from aqueous solution and vacuum-deposited C_60_.^17^ Recently, we showed that solution-processed coplanar Al-doped ZnO diodes fabricated with nanogap electrodes can operate at micro- and millimeter wave frequency bands (intrinsic *f*_cut-off_ > 100 GHz), expanding the application range to 5G and 6G communications.^15^ Furthermore, using the wide-band-gap semiconductor CuSCN as a photoactive material deposited on coplanar nanogap electrodes, we have demonstrated deep UV photodetectors with high responsivity and photosensitivity.^18^

**Figure 2:**
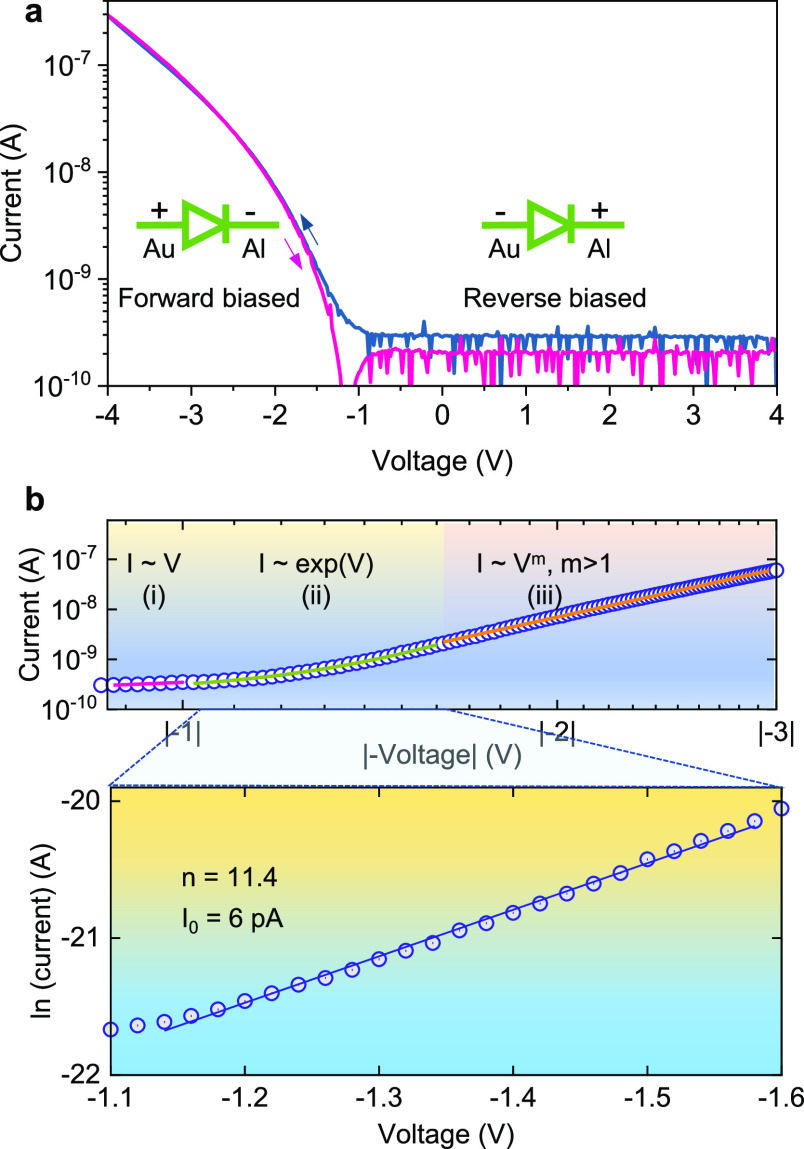
Current–Voltage (*I*–*V*) characteristic of the pristine CuSCN film spin-coated on the top of the prepatterned Al–Au coplanar electrodes in (a) semi-log plot double scans and (b) log–log plot depicting the different transport regimes of the diode under forward bias. The thermionic emission regime is zoomed-in and plotted as ln *I*–*V* to allow calculation of the ideality factor (*n*) and saturation current (*I*_0_) of the p-type diode. Inset: Schematic of the forward and reverse biasing of the diode under test.

CuSCN is a semiconductor of quasi-molecular nature, belonging to the pseudohalide materials class, and is known to exhibit p-type conductivity, mostly owing to Cu vacancies created during material synthesis.^19^ Notwithstanding its attractive optoelectronic properties,^20^ these are very much dependent on the solution processing parameters (e.g., solvent, concentration, spin speed, and thermal annealing), which have been shown to have a direct impact on the film crystallinity, surface roughness, and thickness.^21,22^ An optimized deposition protocol that rendered nanocrystalline films with a thickness comparable to the coplanar electrodes thickness (30–40 nm) resulted in the representative current–voltage (*I*–*V*) characteristic shown in Figure 2a. Current rectification was observed in the negative voltage regime when the Au electrode was biased positive and the Al electrode negative (see insets in Figure 2a), with a rectification ratio at ±4 V equal to 10^3^ and no hysteresis between forward (i.e., biasing toward negative voltage) and reverse scans (Figure S1a). Note that the measured current in the reverse bias is at the same range (∼200 pA) as that of the current we measured for an empty electrode before depositing the CuSCN semiconductor (Figure S1b), indicating that the actual rectification ratio may be even higher. Analysis of the log–log plot of the forward biasing regime of the diode’s *I*–*V* curve reveals three distinct areas (Figure 2b):^23^ (I) an Ohmic region at low voltages (−0.86 to −0.1 V, close to turn-on), where current is proportional to voltage, (II) a thermionic emission region, showing an exponential dependence, indicating the prevalence of thermionic emission of carriers over the barrier, as would be expected in a Schottky diode, and (III) a trap-assisted space-charge-limited current (SCLC) region (>−1.6 V), which obeys a power law relationship (*J*–*V*^*m*^), where *m* = 3.4. SCLC is commonly encountered as the dominant charge transport mechanism in materials with low free carrier density such as dielectrics or semiconductors with wide band gaps or low doping levels.^24^ Pattanasattayavong et al. calculated the unintentional hole doping concentration in CuSCN films (*N*_A_ = 7.2 × 10^17^ cm^–3^), which they attributed to the acceptor-like states, most likely due to the presence of unintentional structural defects and/or chemical impurities derived from solution processing, and proposed the multiple trapping and release model as the dominant mechanism for CuSCN hole transport.^25^ Further analysis of the exponential regime (II) and fitting it to the Shockley diode equation^26^ yield an ideality factor of *n* = 11, a large deviation from the ideal diode (*n* = 1). This can be explained by considering the modified thermionic emission over the inhomogeneous barrier model, which accounts for Schottky barrier inhomogeneities due to spatially distributed fluctuations in the valence band (VB) energy levels or metal work function.^27^ These fluctuations may be due to interface roughness between the Schottky electrode and semiconductor or at metal grain boundaries or due to tunneling currents through interface states or insulating layers (e.g., AlO_*x*_) at the interface,^28^ all of which are likely to coexist given the coplanar nature and the low dimensionality of the Al–Au nanogap electrodes (see the SEM image in Figure 1c).

**Figure 3:**
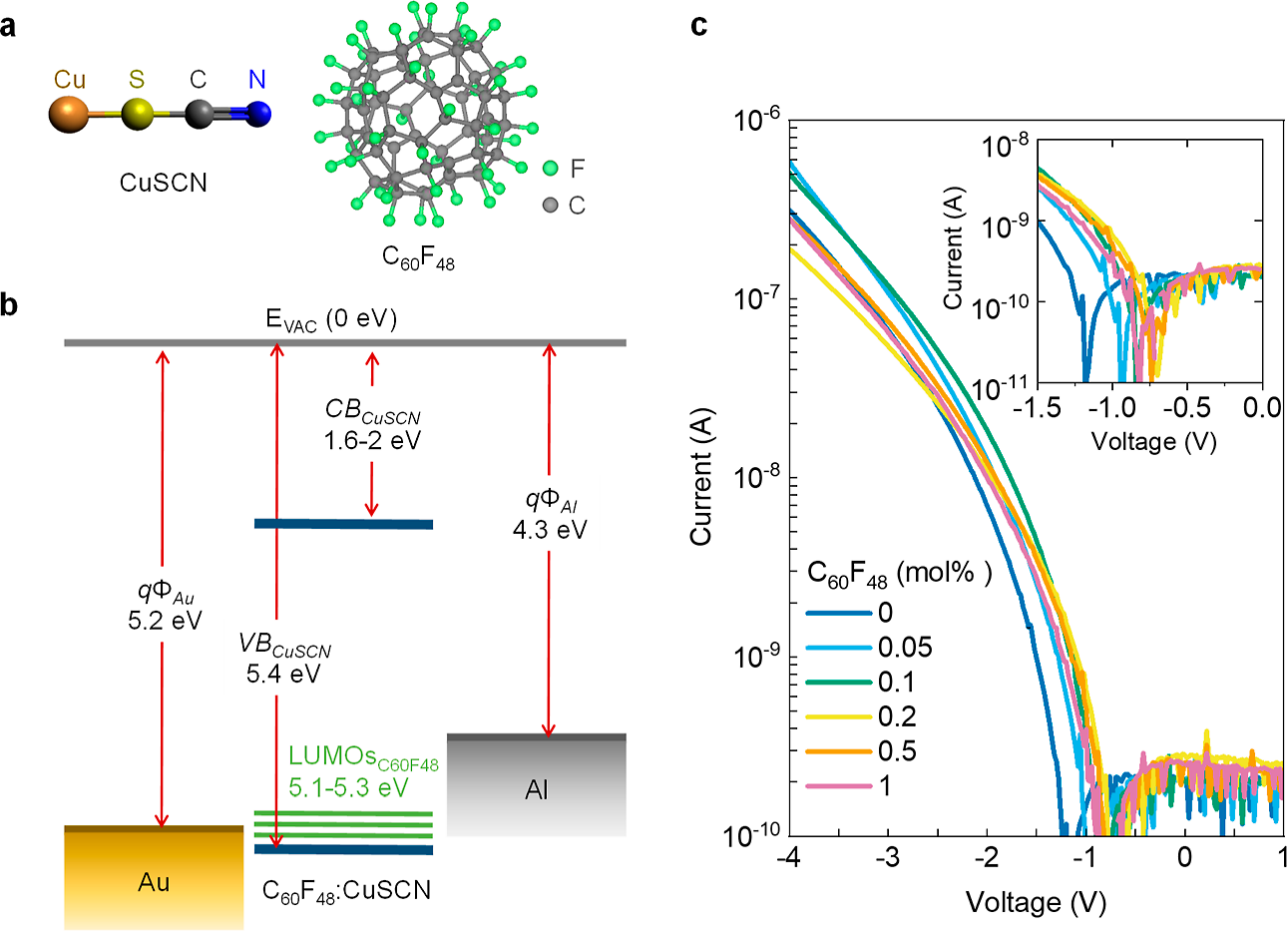
(a) Molecular structures of Cu(I) thiocyanate and of the fluorofullerene dopant C_60_F_48_. (b) Energy band diagram of the Al/CuSCN/Au diode in a flat band configuration, depicting also the acceptor levels introduced by the p-type C_60_F_48_ dopant. The values are derived from ref (29). (c) *I*–*V* characteristics of the undoped (0 mol %) CuSCN film and the CuSCN film doped with 0.05–1 mol % doping of C_60_F_48_. The inset shows a magnified area close to the turn-on voltage of the diodes.

The chemical structures of CuSCN and the p-type dopant C_60_F_48_ are shown in Figure 3a. In the pristine Al/CuSCN/Au diode, Au acts as the Ohmic contact as the energy difference between the Au Fermi level (5.2 eV) and the VB edge of CuSCN (5.4 eV) is relatively small. At the Al/CuSCN interface, however, a Schottky contact is formed due to the work function of Al (4.3 eV) creating a ∼1.1 eV Schottky barrier for holes with the VB edge of CuSCN (Figures 3b and S2). This is contributing to the high rectification ratio and low reverse currents observed with the CuSCN diodes. Doping CuSCN with the molecular p-type fluorofullerene dopant C_60_F_48_ has been previously found to result in the creation of free holes in CuSCN owing to the transfer of electrons from the VB of CuSCN to the lowest unoccupied molecular orbital (LUMO) energy level of C_60_F_48_ (5.3 eV), which lies only 0.1 eV above the VB edge of CuSCN.^29^ The energetics of the Schottky and Ohmic interfaces including the dopant LUMO energy levels are illustrated in Figure 3b, while the injection of majority carriers (holes) under forward and reverse biasing of the diode is depicted in Figure S2.

**Figure 4:**
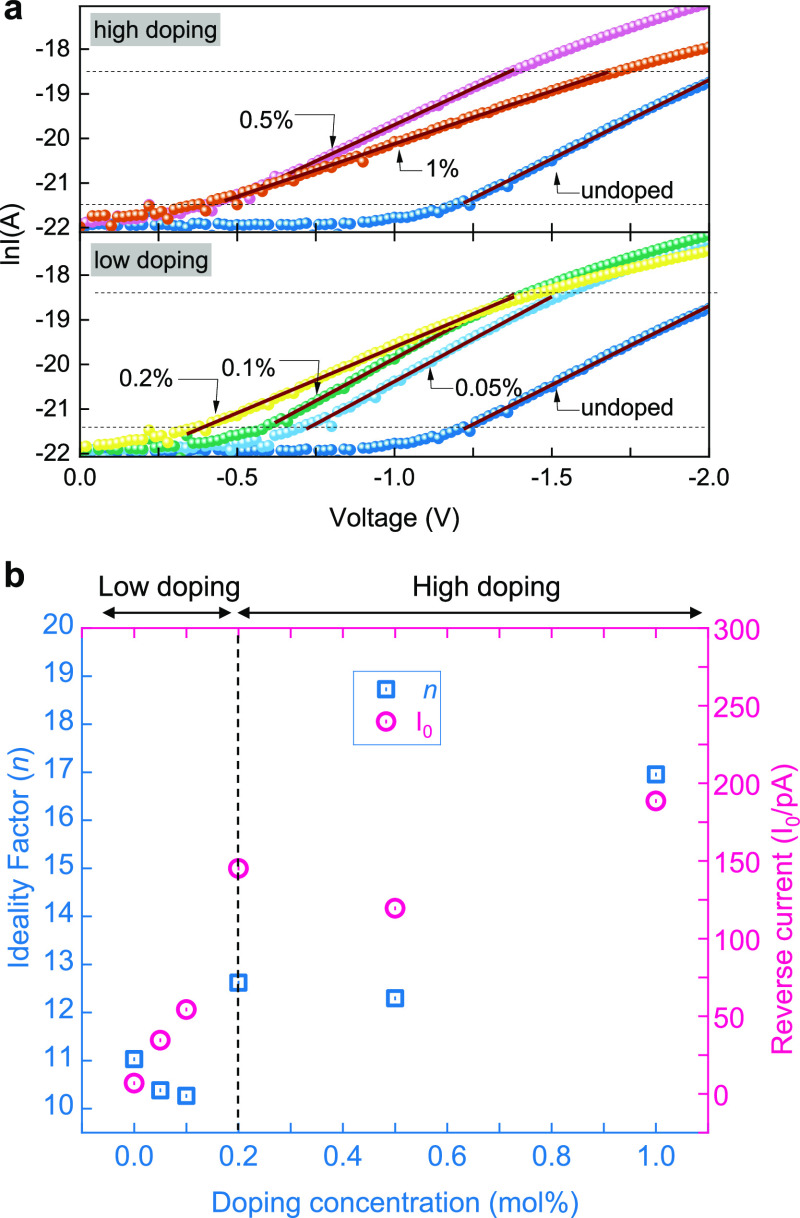
(a) ln *I*–*V* plots of the forward biasing regime of 0–0.2 mol % (low) and 0.5–1 mol % (high) doping levels of CuSCN films and linear fits to the thermionic emission regions (depicted with dashed horizontal lines) used for the extraction of the ideality factor (*n*) and saturation (reverse) currents (*I*_0_), summarized in Supporting Information Table S1 and in (b) for both low and highly doped films.

The *I*–*V* characteristics of pristine and C_60_F_48_-doped CuSCN diodes are shown in Figure 3c and are characterized by high asymmetry. The doping concentrations ranged from 0 (undoped) to 1 mol %. The diode properties are initially improved as the doping concentration increases up to 0.2–0.5 mol % and the turn-on voltage of the diode shifts toward lower (less negative) values. This is better illustrated in the inset of Figure 3c. Further analysis of the *I*–*V* characteristics is presented in Figure 4a, which depicts the linear fit of the forward biasing region of the *I*–*V* curves at the thermionic emission regime of the undoped, low doped (0.05, 0.1, and 0.2%), and highly doped (0.5 and 1%) diodes in a ln *I*–*V* plot. The results of the fitting to the Shockley diode equation are shown in the Supporting Information Table S1 and summarized in Figure 4b. It is evident that the diode ideality factor is reduced for low doping concentrations (up to 0.1 mol %), whereas it begins to increase again when the doping increases further up to 1 mol %, and the results obtained for doping levels >0.2 mol % do not follow a monotonic increase. On the other hand, the reverse current generally increases with the doping concentration. A plausible explanation for these observations can be proposed by considering the effect of doping on both the film morphology and the energetics of the Schottky diode device. An atomic force microscopy study that has been previously published by our group^29^ showed that the addition of even small amounts of C_60_F_48_ forms ellipsoidal (platelet-like) grains with a typical diameter of ≈20 nm, a value which is comparable to the nanogap channel length. Increased dopant concentration results in the formation of larger crystallites that may obstruct the material from filling the nanogap, negatively affecting the charge transport between the coplanar electrodes. This is manifested in the lower forward current and deterioration of the diode characteristics upon a higher level of doping. However, in the low-doped devices where this effect is not prominent, the increased forward current and the shift in the *I*–*V* toward lower voltages that were observed in Figure 3c are an indication of the increased conductivity due to the passivation of existing traps in CuSCN by C_60_F_48_. On the other hand, the formation of acceptor levels close to the CuSCN VB edge reduces the barrier height, and this may be the reason of the small increase in reverse current as the dopant concentration increases. It should be noted that the diodes showed good stability after storage in air for 60 min with minimal reduction in the forward current and no increase in the reverse current (Figure S3). Similar stability was observed in flexible undoped CuSCN thin film transistors,^12^ while C_60_F_48_ is expected to further contribute to ambient stability (especially toward water uptake in the film) due to its inherent hydrophobicity owing to its high fluorine content, as compared, for example, to pure C_60_ fullerenes.^30^

**Figure 5:**
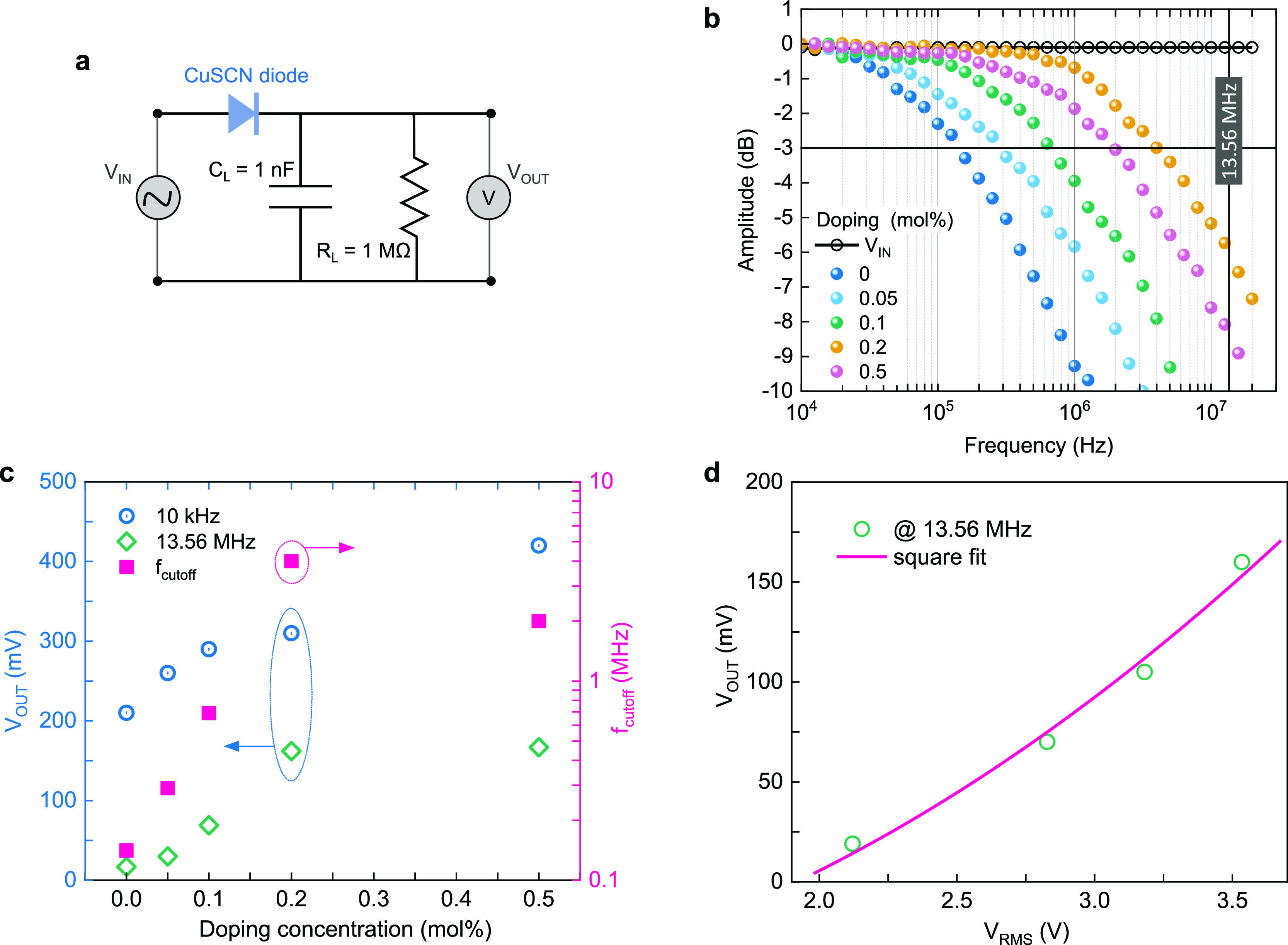
(a) Half-wave rectifier circuitry comprising a 1 nF load capacitor (*C*_L_) and a 1 MΩ load resistor (*R*_L_) mounted directly onto the measurement micromanipulator for measuring the DC output voltage from the coplanar CuSCN diodes. (b) *V*_OUT_ amplitude (in dB) as a function of frequency depicting the *f*_cutoff_ for the undoped and C_60_F_48_-doped CuSCN diodes at −3 dB for *V*_IN_ = ±5 V (corresponding to *V*_RMS_ = 3.53 V). (c) *V*_OUT_ calculated at 10 kHz and 13.56 MHz and *f*_cutoff_ values as a function of the doping concentration. (d) *V*_OUT_ at the commercially relevant RFID frequency of 13.56 MHz vs varying input *V*_RMS_ signals.

What renders Schottky diodes particularly attractive for high-frequency applications is their fast response time due to the presence of majority carriers (unipolar devices). To explore the high-frequency response of the pristine and C_60_F_48_-doped CuSCN diodes, we constructed a half-wave rectifier circuit as shown in Figure 5a. The circuit consists of a 1 nF load capacitor and a 1 MΩ load resistor to obtain a stable DC output voltage (*V*_OUT_) following the rectification of the AC input signal (*V*_IN_) by the CuSCN diode. Figure 5b shows the dependence of *V*_OUT_ as a function of *V*_IN_ frequency in a decibel scale. The cutoff frequency (*f*_cutoff_) is defined as the frequency at −3 dB, namely, the frequency at which the output voltage of the diode drops to 1/√2 of its peak value at low frequency. It can be seen that *f*_cutoff_ increases gradually from 0.14 to 4 MHz as the doping level increases up to 0.2 mol % and then starts to decrease (2 MHz for 0.5 mol % doping), which is in accordance with the DC characterization results. Figure 5c shows the *V*_OUT_ and *f*_cutoff_ as a function of the doping level for an AC peak-to-peak voltage (*V*_PP_) of 10 V (±5 V). The highest *f*_cutoff_ of 4 MHz is obtained for the 0.2 mol % C_60_F_48_-doped CuSCN diode and is shown in Figure S4, while statistical data of the *f*_cutoff_ and *V*_OUT_ measured for six distinct devices at each doping level are given in Figure S5. For the same device, the output voltage at 13.56 MHz was plotted as a function of the input root mean square voltage (*V*_RMS_ = 1/2√2 × *V*_PP_), and a square law fit was obtained, showcasing the good rectifying properties of these diodes (Figure 5d). The low-frequency (10 kHz) *V*_OUT_ was 310 mV for this diode, whereas at the commercially relevant frequency of 13.56 MHz, the diode still outputs 162 mV, proving that it can be of use in high-frequency RFID tags and, more specifically, in targeted applications with low power demands. This corresponds to an efficiency of about 10^–5^% (*P*_OUT_/*P*_IN_, where *P*_OUT_ is calculated from the *V*_OUT_ over the 1 MΩ load resistor). The very low energy harvesting efficiency could be attributed to the parasitic series resistance of the diode, *R*_S_, since the RF energy coupled into *R*_S_ is lost as heat and does not contribute to the rectified output of the diode. Careful design that minimizes the bond wire and lead frame resistance on the final rectifier circuitry, as compared to that of the bulk experimental setup we used here, could significantly improve this. Interestingly, the output voltage of the 0.5 mol % doped diode is higher despite the lower *f*_cutoff_ and slightly deteriorated diode characteristics. This is an indication for improved transport at higher frequencies owing possibly to faster kinetics at the trapping sites introduced by the acceptor levels of the dopant.

An example of the input AC waveform and the rectified *V*_OUT_ is shown in Figure S6. The ripple that can be seen in the output voltage is probably due to the mismatch of the load capacitor with the capacitance of the RF diode. We can minimize this ripple by keeping the period of the input signal below the load RC time constant, τ_RC_, something which holds for relatively high frequencies but can be problematic at kilohertz frequencies. Additionally, the load capacitance should generally be much larger than the capacitance of the diode (typically, for these diodes, capacitance is <1 pF); however, this depends on the diode area and should be taken into account when different diode widths are implemented. The load resistor over which the output voltage is measured could also be modified to control the magnitude of the output voltage.^31^ Moreover, parasitic junction capacitance of the diode, *C*_J_, could be minimized by controlling the geometrical characteristics of the diode. We have showed in our previous publication how increasing the diode width can increase the output voltage at the expense of a lower cutoff frequency.^15^ In this paper, we show how to decrease the high resistivity of the semiconductor material by doping; however, it should be noted that the voltage drop can be further reduced by improving the currently nonideal metal–semiconductor contacts, for example, via applying specific treatments or SAMs to the electrodes.^13,32^ Finally, the design of the rectifier topology is of critical importance, as integration of single diodes in full-wave or bridge rectifiers or even voltage multiplier circuits can tune the performance accordingly to fit the requirements of the targeted applications.^33^

## Conclusions

We demonstrated Schottky diodes based on the abundant nontoxic p-type semiconductor CuSCN, processed from solution at low temperature, and nanogap coplanar electrodes fabricated using a-Lith. A molecular doping strategy was adopted using the p-type dopant C_60_F_48_ at an optimized ratio to the CuSCN solution. The fabricated Schottky diodes showed a high rectification ratio (>1000) and a low reverse current (200 pA) in DC, and the effect of the dopant concentration in the current–voltage characteristics was analyzed and discussed. It was found that relatively low doping levels (<0.2 mol %) improve the diode properties owing to the C_60_F_48_ providing electrons close to the CuSCN VB (acceptor levels), thus reducing the barrier height and improving injection and transport without inducing a major increase in the leakage current. The high-frequency rectifiers fabricated with these diodes showed cutoff frequencies in the megahertz regime and output voltages in the order of hundreds of millivolts (*V*_in_ = ±5 V). These results compare well with that of recently published nanoscale rectifiers based on copper phthalocyanine (CuPc) molecular diodes with thickness of 4.5–15 nm, showing a rectification ratio of up to 500 and *f*_cutoff_ ≈ 10 MHz, which, however, were fabricated using more complicated and less prone to industrial upscale methods.^34^ Thus, with this work, we show that p-type rectifiers can be fabricated with inexpensive materials and using high-throughput patterning techniques compatible with flexible electronics and implemented in low-cost disposable RFID tags or NFC devices for smart packaging, medical devices and patches, and the internet of things.

## Supporting Information Available

Characteristics of diodes upon doping, additional *I*–*V* curves of the empty and CuSCN-filled nanogap electrodes, energy level diagrams depicting injection at forward and reverse biasing, diode stability in air, and high-frequency supplementary and statistical performance data (PDF)
