# Supplementary material for: Radiofrequency Schottky Diodes Based on p-Doped Copper(I) Thiocyanate (CuSCN)
Source: ACS Appl Mater Interfaces. 2022 Jun 1;14(26):29993–9. doi: 10.1021/acsami.1c22856 (PMC9264318; doi:10.1021/acsami.1c22856)
Supplement: Supplementary file 1 — am1c22856_si_001.pdf [file am1c22856_si_001.pdf]

Supporting Information

Radio-Frequency Schottky Diodes Based on p-Doped Copper(I) Thiocyanate (CuSCN)

Dimitra G. Georgiadou,[*,1,2] *Nilushi Wijeyasinghe,*[2] *Olga Solomeshch,*[3] *Nir Tessler,*[3] *Thomas D. Anthopoulos*[*,2,4]

[1]Electronics and Computer Science, University of Southampton, Highfield Campus, Southampton SO17 1BJ, United Kingdom.

[2]Department of Physics, Imperial College London, Prince Consort Road, South Kensington, London SW7 2AZ, United Kingdom.

[3]The Zisapel Nano-Electronic Center, Department of Electrical Engineering, Technion-Israel Institute of Technology, Haifa 3200, Israel

[4]King Abdullah University of Science and Technology (KAUST), Division of Physical Sciences and Engineering, Thuwal 23955-6900, Saudi Arabia.

*E-mails: d.georgiadou@soton.ac.uk and thomas.anthopoulos@kaust.edu.sa

Supplementary Table

Table S1. Ideality factor (n) and reverse current (I_0) derived from fitting to the Shockley equation.

**Semiconducting film**	**Ideality Factor (n)**	**Reverse current (I_0) [A]**
**pristine CuSCN**	10.9	6.5×10^{-12}
**0.05 mol% $C_{60}F_{48}$**	10.1	3.2×10^{-11}
**0.1 mol% $C_{60}F_{48}$**	10.1	5.4×10^{-11}
**0.2 mol% $C_{60}F_{48}$**	13.1	1.6×10^{-10}
**0.5 mol% $C_{60}F_{48}$**	11.8	1.1×10^{-10}
**1 mol% $C_{60}F_{48}$**	16.3	1.7×10^{-10}

Supplementary Figures

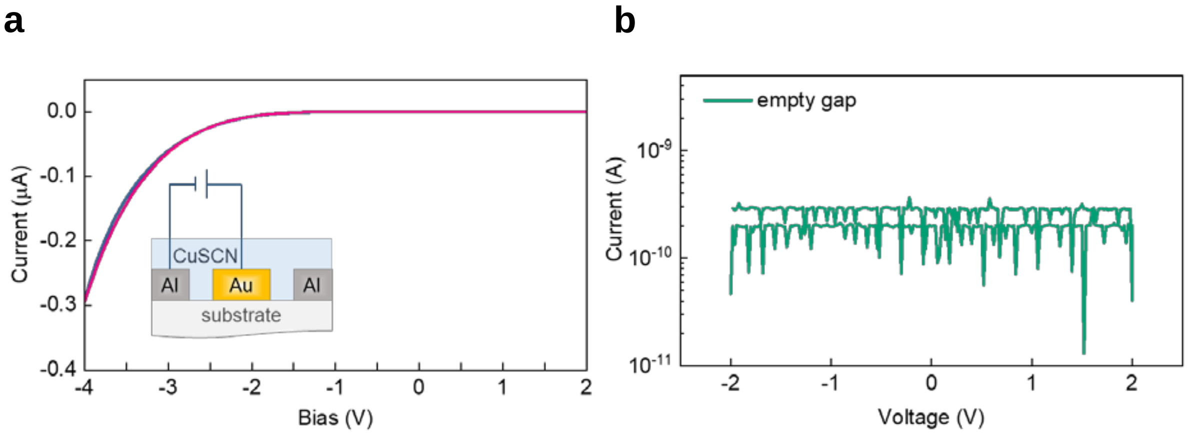

Supplementary Figure S1. a) Linear I-V characteristic for the CuSCN diode showing minimal hysteresis. Inset: Schematic depicting the voltage applied to the coplanar nanogap electrodes in the forward biasing regime. b) Semi-log I-V characteristic of the empty nanogap, before depositing the CuSCN semiconductor, applying the same measurement parameters in the SMU as in the CuSCN diodes.

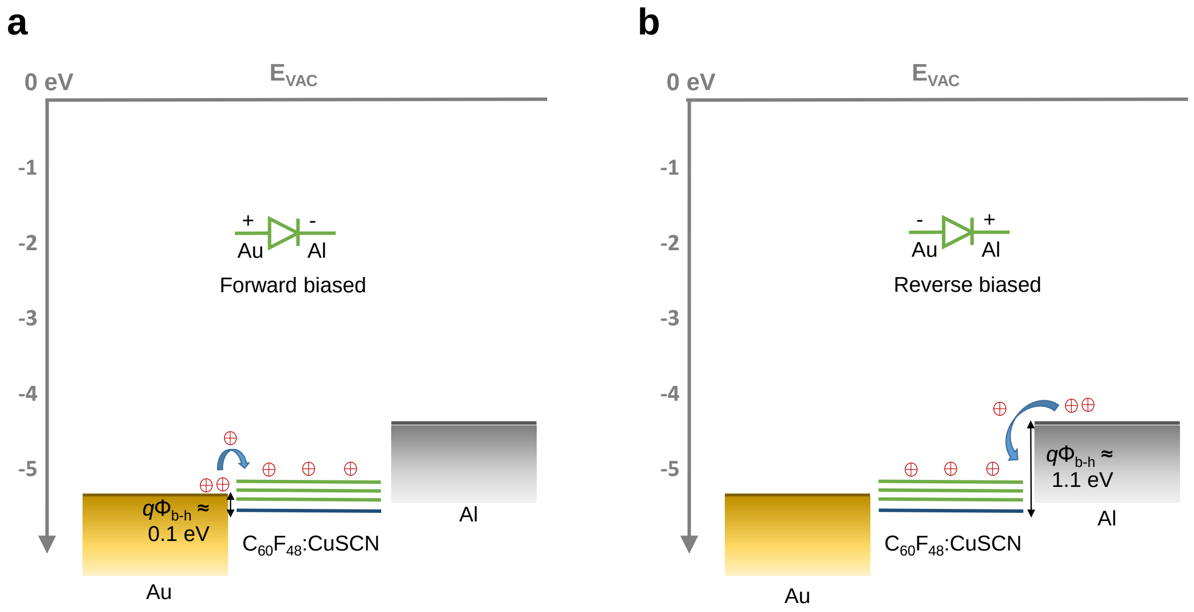

Supplementary Figure S2. Energy level diagrams in a flat band configuration illustrating the majority carrier (hole) injection during a) forward and b) reverse basing of the diode. The respective injection barrier heights are also depicted in each case.

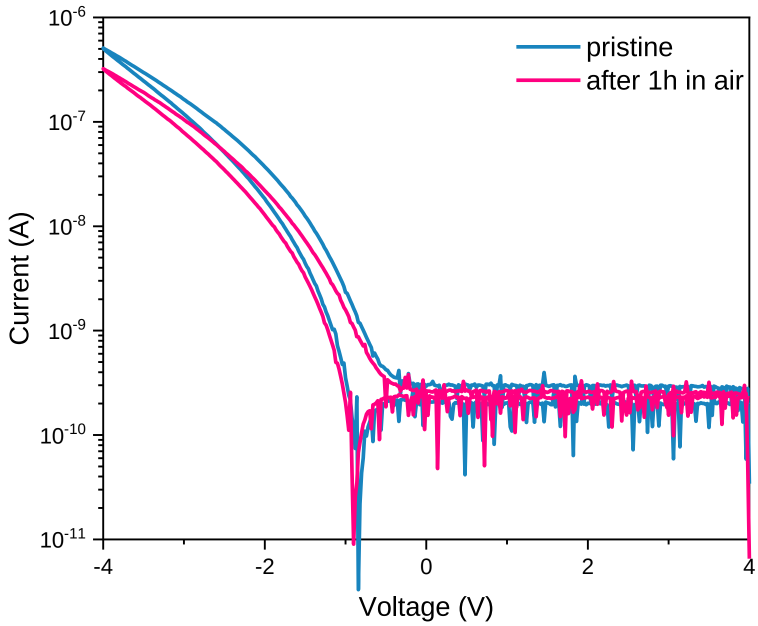

Supplementary Figure S3. Semi-log I-V characteristic for the 0.2 mol% $C_{60}F_{48}$ doped CuSCN diode measured in air, right after fabrication (blue) and after 1h in air (pink).

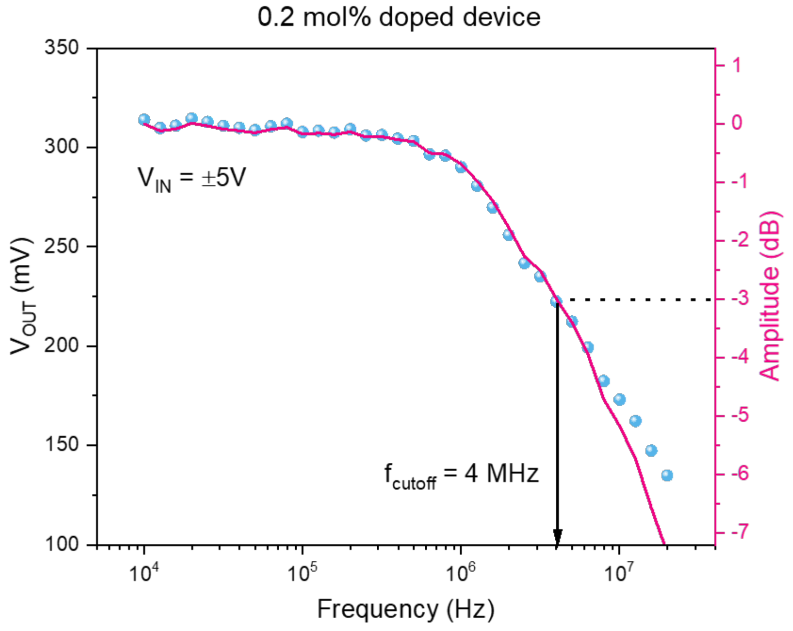

Supplementary Figure S4. Output voltage vs frequency curve for the 0.2 mol% $C_{60}F_{48}$ doped CuSCN diode, depicting a cutoff frequency of 4 MHz at -3dB.

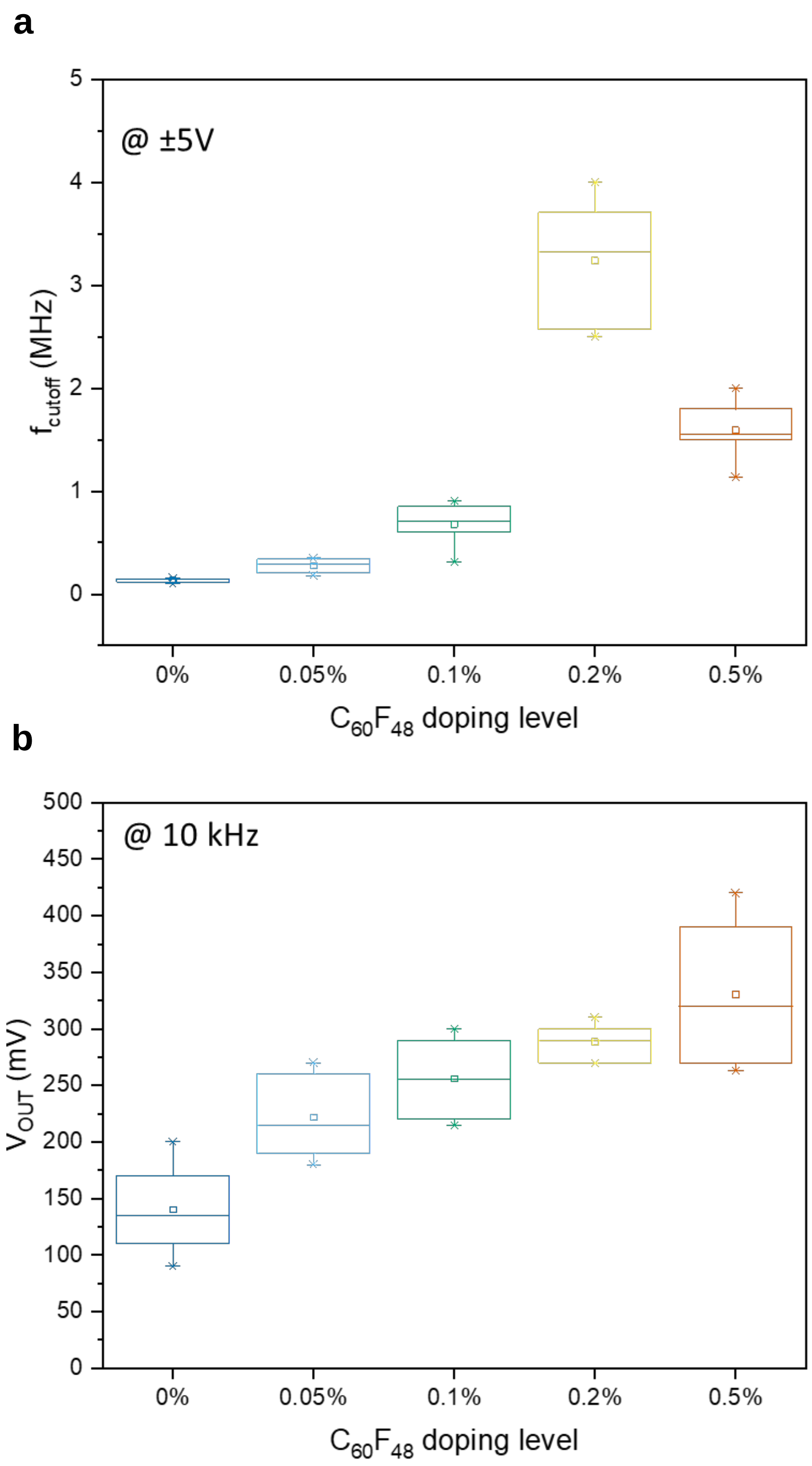

Supplementary Figure S5. Statistical data of the (a) cutoff frequency (at V_{pp} = 10 V) and (b) output voltage (at 10 kHz) as a function of $C_{60}F_{48}$ doping level measured for 6 distinct CuSCN-based diode devices.

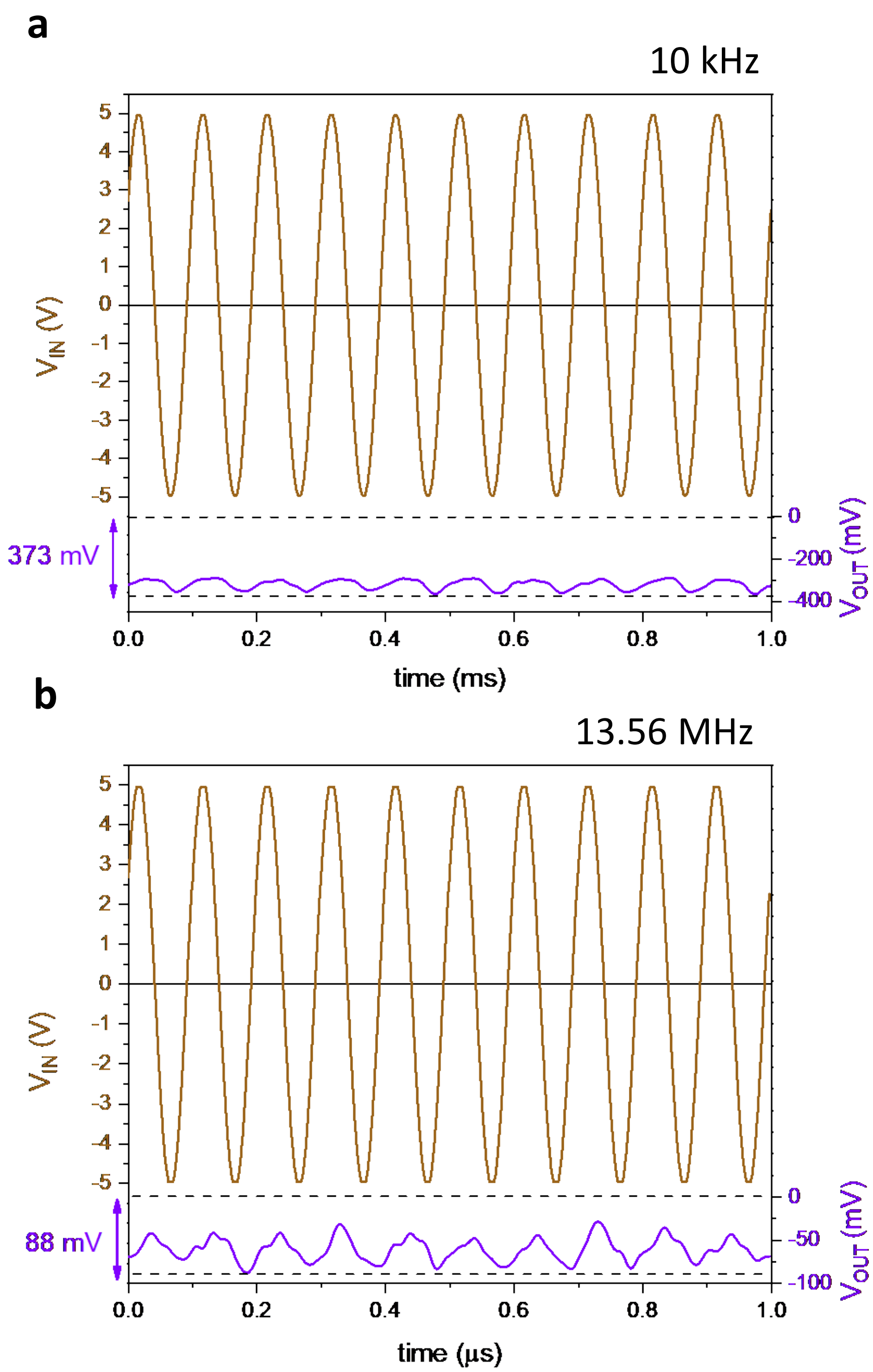

Supplementary Figure S6. AC input waveform and the rectified V_{OUT} at (a) 10 kHz and (b) 13.56 MHz for a 0.5 mol% $C_{60}F_{48}$ doped CuSCN diode.
